# The structure of the annual migratory flight activity in a songbird

**DOI:** 10.1098/rspb.2025.0958

**Published:** 2025-10-15

**Authors:** Sissel Sjöberg, Pablo Macías-Torres, Arne Andersson, Johan Bäckman, Kasper Thorup, Anders Peter Tøttrup, Thomas Alerstam

**Affiliations:** ^1^ Department of Biology, Lund University, Lund, Skåne County SE-223 62, Sweden; ^2^ Center for Macroecology, Evolution and Climate, Globe Institute, University of Copenhagen, Copenhagen DK-2100, Denmark; ^3^ Natural History Museum Denmark, University of Copenhagen, Copenhagen DK-1350, Denmark

**Keywords:** songbirds, bird migration, migration programme, biologging, accelerometer, flight activity, circannual programme, *Lanius collurio*

## Abstract

Migratory songbirds have an internal circannual genetic programme that controls the timing and extent of migratory flight activity, as demonstrated by experiments with birds held in cages. We used multisensor data loggers to record the timing and duration of all migratory flights during the annual cycle of 15 free-living individuals of red-backed shrikes *Lanius collurio*. Annual actograms unexpectedly revealed that the nocturnal migratory flights of the shrikes were organized in a highly structured way, with flights aggregated into segments that could be readily identified for all individuals, showing low variability and thus high consistency between individuals. These results suggest that the execution of migratory flights is under a high degree of control according to a rather detailed internal travelling plan for the annual migration cycle. Potentially, the control of migratory flight under natural conditions depends on a complex feedback process where external cues associated with the geographic, temporal and nutritional situation of the bird are required for the internal programme to properly regulate the successive segmental flight steps of the migratory journey. This would mean that the internal/genetic programme for control of bird migration is much more dynamic and complex than hereto assumed.

## 1. Introduction

### (a) An endogenous circannual programme for migratory flight activity

Naturalists had already observed and reported 200 years ago that migratory songbirds, when kept in cages for long periods of time (several months or years), showed migratory restlessness (or ‘Zugunruhe’) during the autumn and spring migratory seasons [1–3]. This indicates that migratory birds have an internal ‘instinct’ for undertaking their migratory flights and completing their migratory journeys at the proper times in autumn and spring. This internal driving force for migratory flight activity was not explored in a systematic way until more than a hundred years later, when experimental techniques for recording and measuring activity of caged birds (with the results showing up as actograms on a daily and seasonal basis) were worked out by Wagner [4] and Palmgren [5]. A major breakthrough took place with the demonstrations and analyses of endogenous circannual rhythms in different species of long- and short-distance migrants by Gwinner [6,7] and Berthold [8]. Gwinner’s [6] experiments in which the activity of birds that lived in cages for several years was continuously recorded revealed that the ‘free-running’ rhythms of migratory restlessness under constant external conditions deviate slightly but consistently from a 12 month cycle. It turned out to be the external influence of the seasonally changing day length (photoperiod) that synchronizes these rhythms with the 12 months of the year.

Long-term monitoring of migratory restlessness in many different species of migrants kept in cages by using activity registration revealed that migratory birds have an innate programme that defines the autumn and spring migratory periods (reviewed in [9,10]). While the nocturnal migratory restlessness of caged migrants seems to reflect the normal onset and temporal course of autumn migration and the beginning of spring migration, there is often a discrepancy between the termination of spring migration in the wild and among caged migrants. Among caged birds, high levels of restlessness often continue into the summer breeding season. The reason for this discrepancy is unknown but may be related to the inability of caged birds to perform reproductive activities [11].

The internal migratory programme seems to regulate not only the timing of migration but also the total distance of the migratory journey. There is a clear positive correlation between total migratory activity during the autumn migratory period (measured as hours of nocturnal migratory restlessness) and the total migration distance, suggesting that the internal programme controls migration distance by producing just enough migratory flight activity for the birds to reach their specific main non-breeding destinations [9,10]. The seasonal total of migratory restlessness, thus presumably regulating the total distance of migration, is a trait with high heritability, as demonstrated by crossbreeding experiments (hybrids between individuals from short- and long-distance migratory populations show intermediate levels of seasonal total migratory restlessness) and by artificial selection experiments [10].

In addition to the endogenous regulation of the timing and distance of migration, it has also been demonstrated that the birds’ orientation is under internal control, with changing preferred migratory flight courses during the circannual cycle [12]. There are also endogenous circannual cycles for regulation of fuel deposition and moult in relation to the migration periods. There are other components of migratory behaviour that must also be genetically preprogrammed to allow an inexperienced young bird to complete a long migratory journey entirely on its own. For example, instructions about changes in stopover duration, fuel deposition and flight step length at barriers (such as deserts or oceans) that the birds cross by non-stop or prolonged flights are probably included in their internal migratory programme (see also more recent reviews [13–15]).

Is it possible that the internal programme for migratory restlessness regulates not only the seasonal total of migratory flight activity, but also, to some degree, controls the number and distribution of migratory flights throughout the seasonal migration period? This would mean that a more detailed travelling plan for the migratory journey would be under control of the internal genetic migration programme. Although seasonal distribution curves of migratory restlessness often deviate from a uniform unimodal pattern, showing different degrees of skewness and multimodality, it seems that this variation in the circannual actograms of caged migrants has not been analysed more closely. It has been implicitly assumed that, during the migratory seasons, migrants primarily adapt their flight steps and stopover periods according to external conditions like the weather, winds and local fuel availability, according to the principles of optimal bird migration (e.g. [16–19]). Given the great potential variability in weather and local fuel deposition conditions between years, regions and time periods, one would predict that the annual pattern of flight and stopover periods by which a seasonal migratory journey is completed would vary considerably between and within individuals.

In this study, we used multisensor data loggers [20] to track the annual flight activity of free-flying red-backed shrikes *Lanius collurio*. The population of red-backed shrikes that we study is known to perform an annual loop migration of approximately (*ca*) 20 000 km, with multiple longer staging periods between their breeding area in Scandinavia and main wintering area in southern Africa [21]. Previous studies of the migration of this red-back shrike population have shown low inter-individual variability in routes and timing of migration [21–23]. In addition, by using multisensor data loggers, we have previously described the annual daytime activity in this population in detail (a study that is also to a large degree based on data from the same individuals that are included in this study [22]). Like many insectivorous songbirds, the red-backed shrike is assumed to travel solitarily, normally performing its migratory flights during the nights (but see [24,25]). Such solitary migrants are expected to be guided to their migratory destination entirely by their internal migratory programme during their first journey as juveniles, when they lack all experience of conditions along the travel routes. They are expected to learn from the first journey, so that subsequent journeys, which they perform as adults, are likely to be more efficient.

### (b) Objective of this study

**Figure 1.**
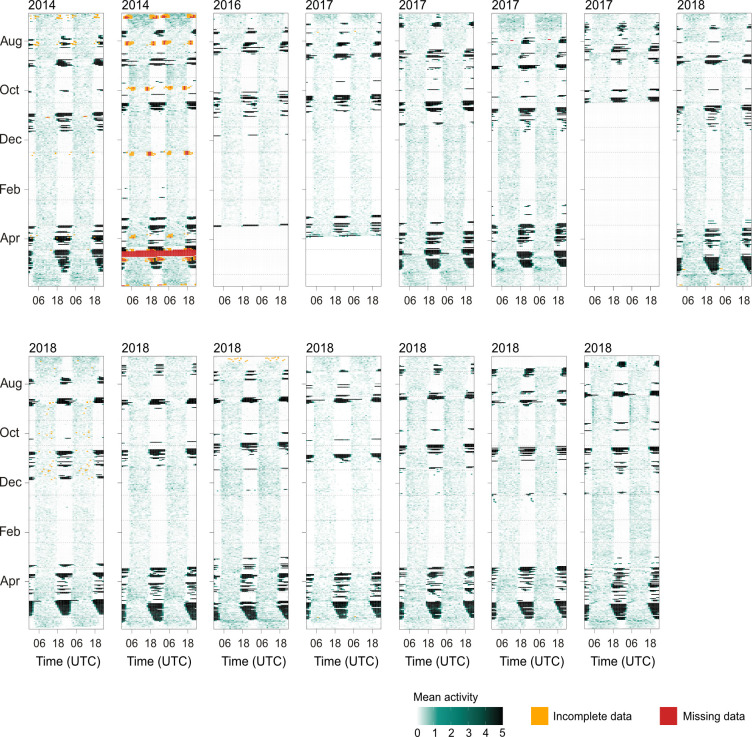
Full annual activity patterns (including odd flights outside the migratory flight segments; see §2b) based on the raw accelerometer data of all red-backed shrikes included in the study illustrated as double-plotted actograms. Average activity per hour as measured by the accelerometer (white = no activity; green = medium activity, e.g. foraging or territorial behaviour; black = high activity/sustained flight; see text). The vertical axes represent the succession of days for one migratory cycle, from 15 July in the year the bird was equipped with the data logger until 15 June in the successive year. Each horizontal row represents accelerometer data from 2 consecutive days, where the second day, on the right, is repeated as the first day on the following row to facilitate full visualization of diurnal and nocturnal activity patterns. Sustained high activity for >30 min was classified as a flight (see §2b). Yellow and red indicate incomplete and missing data, respectively.

**Figure 2.**
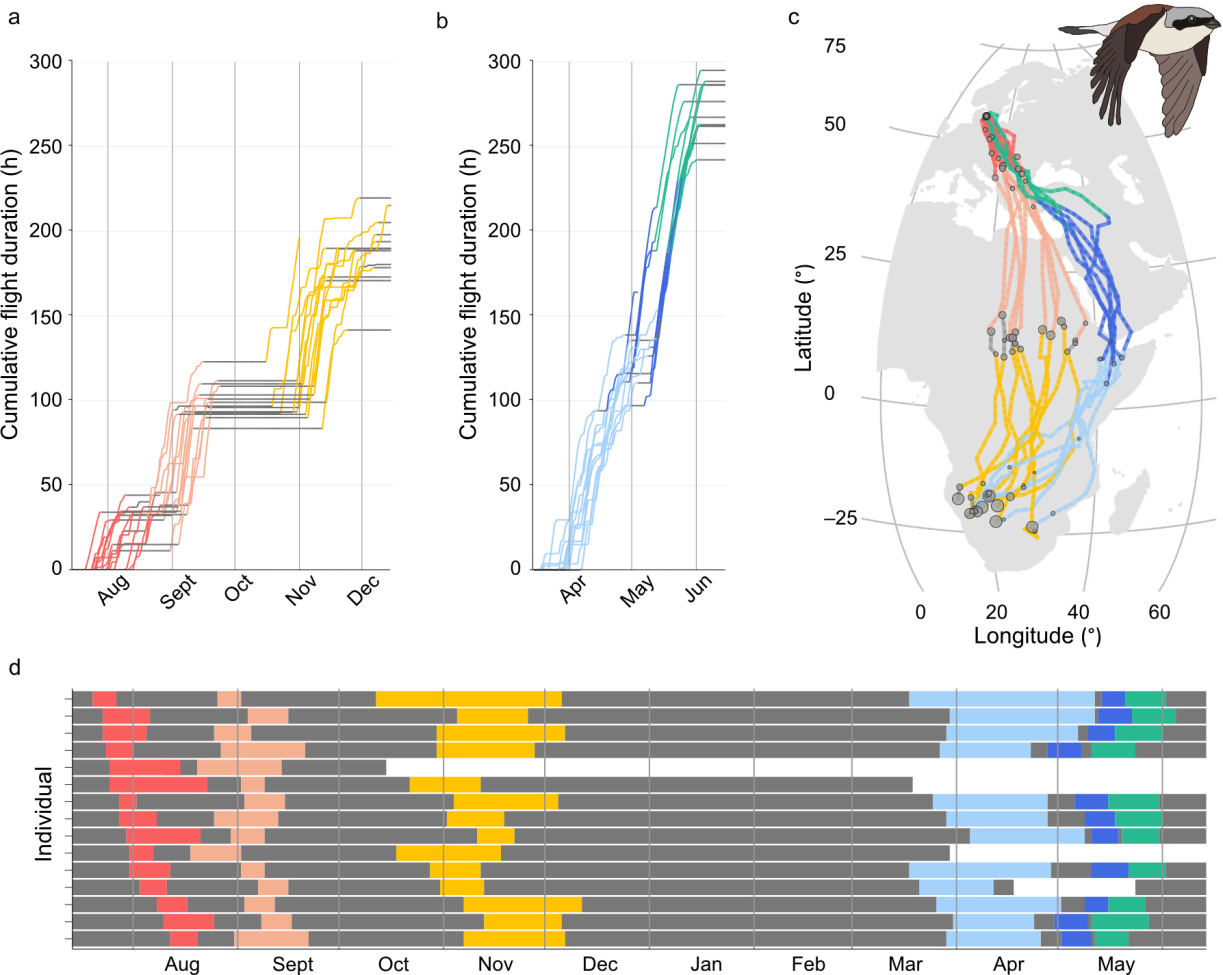
Cumulative flight duration (number of flight hours) during (a) autumn and (b) spring migration in red-backed shrikes migrating between their breeding area in Denmark and their wintering areas in southern Africa (each line illustrates an individual; no odd flights included, see §2b). (c) Illustration of the estimated most likely migratory routes of the 9 individuals where the barometric pressure data allowed for estimation of their geographic positions (each line illustrates an individual; grey circles indicate stopover sites where the birds stayed longer than 5 days; size of circle corresponds to duration of stay. The map was generated with the maps package for R; [26]), (d) Phenology of the different flight segments per individual. Colours in all panels represent flight segments identified from clustered flights in the actograms in figure 1 (segment 1 = red, segment 2 = pink, segment 3 = yellow, segment 4 = light blue, segment 5 = dark blue, segment 6 = green; grey represents stationary periods).

**Figure 3.**
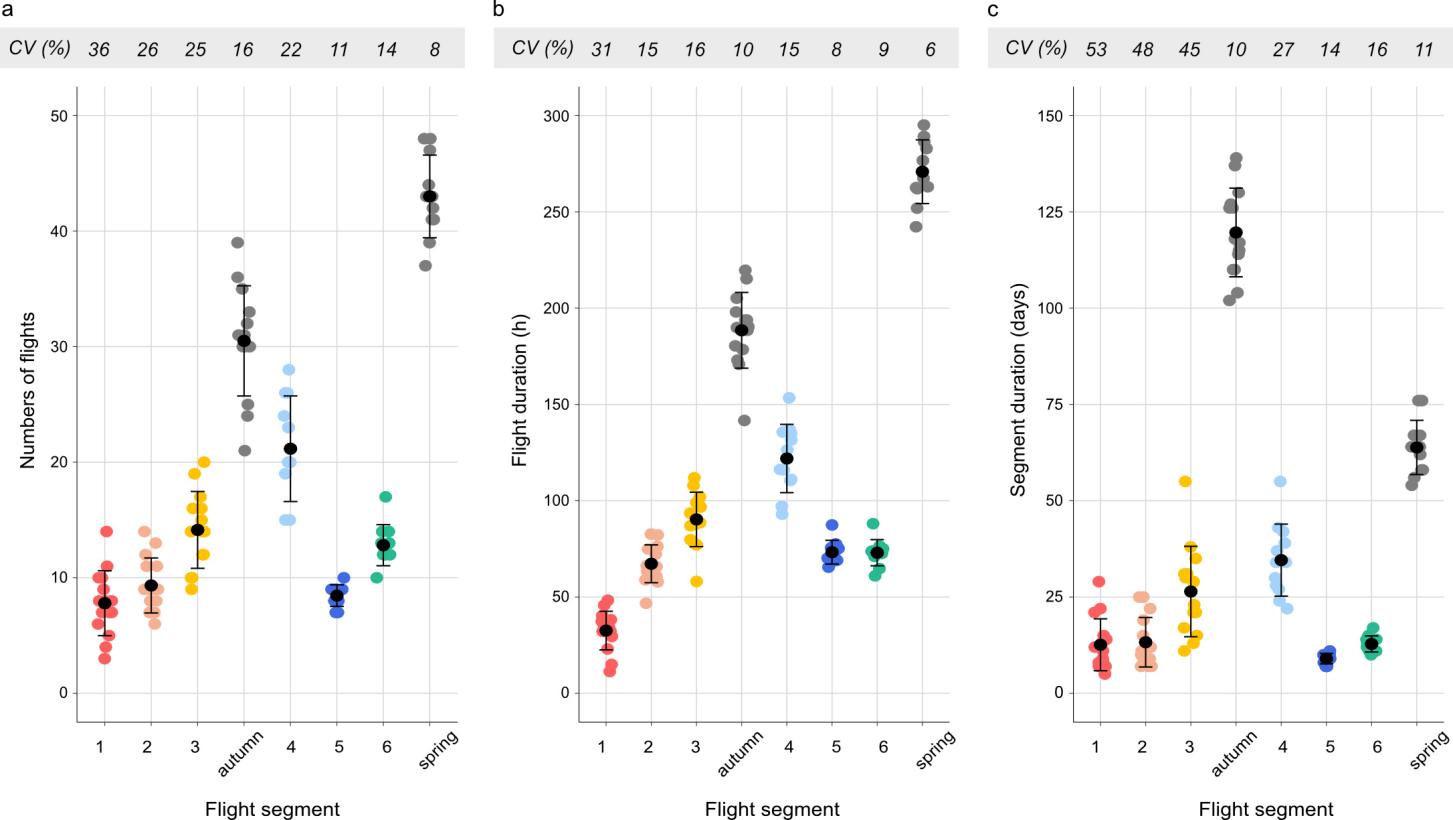
Variation in (a) numbers of flights, (b) flight duration (in hours) and (c) segment duration (in days) within each flight segment and migratory season between the different individuals. Coloured points represent individuals within a flight segment (segment 1 = red, segment 2 = pink, segment 3 = yellow, segment 4 = light blue, segment 5 = dark blue, segment 6 = green), grey points represent individuals within a migratory season and black points and bars indicate mean ± s.d. The coefficient of variation (CV) per segment and migratory season is given at the top of the figures.

Not until very recently has it become possible to monitor the flight activity (with high time resolution; 5 min) of long-distance migratory songbirds in the wild throughout the annual cycle, by way of multisensor data loggers with miniature accelerometers [20]. Here, we analysed annual actograms from 15 free-flying red-backed shrikes to reveal the characteristics and variation in their migratory flight patterns (figures 1–3, table 1). Specifically, we describe and analyse how the distribution of migratory flight activity was highly consistent between individuals and organized into characteristic segments of the migratory journey that could be identified for all individuals. From the actograms, we identified flight segments and the number and duration of the migratory flights, and these data were analysed with respect to the variation between individuals. Furthermore, when possible, we used barometric pressure data to document the geographic extent of the different flight segments. These results are discussed in relation to the endogenous seasonal rhythm of migratory restlessness among juvenile red-backed shrikes (kept in cages under constant conditions) as recorded by Gwinner & Biebach [27]. We think that this analysis throws new light on the relative importance of internal and external factors for the observed flight itineraries for long-distance migrations of songbirds.

**Table 1.** Basic descriptives of the annual migratory flight activity in red-backed shrikes migrating from breeding areas in southern Scandinavia to non-breeding areas in southern Africa using a loop migration of *ca* 20 000 km. All descriptives are given as mean ± s.d./*n* (where *n* = sample size of individuals). Distances are approximated from the tracks of the birds where it was possible to use pressure data for positioning. Odd flights are not included (see §2b).

segment	start date (date ± days)	end date (date ± days)	no. of flights	duration of flight segment (days)	total flight duration (hours)	average duration per flight (hours)	approximate distance (km)
1	31 Jul ± 7.0/15	12 Aug ± 8.7/15	7.8 ± 2.8/15	12.6 ± 6.7/15	32.6 ± 10.0/15	4.2 ± 1.7 /15 Max 8.2	1420
2	31 Aug ± 6.2/15	13 Sep ± 6.1/15	9.3 ± 2.4/15	13.3 ± 6.4/15	67.3 ± 9.8/15	7.2 ± 3.8/15 Max 17.2	3560
3	1 Nov ± 9.0/15	28 Nov ± 10.6/14	14.1 ± 3.3/14	26.4 ± 11.8/14	90.3 ± 14.1/14	6.4 ± 3.4/14 Max 11.0	4140
autumn	31 Jul ± 7.0/15	28 Nov ± 10.6/14	30.5 ± 4.8/14	119.6 ± 11.5/14	188.5 ± 19.7/14	6.2 ± 3.4/14 Max 17.2	9120
4	28 Mar ± 5.5/14	2 May ± 8.8/12	21.2 ± 4.6/12	34.6 ± 9.4/12	121.9 ± 17.7/12	5.8 ± 3.2/12 Max 10.8	4680
5	8 May ± 7.6/12	19 May ± 5.0/11	8.5 ± 0.9/11	9.0 ± 1.3/11	73.3 ± 6.2/11	8.7 ± 2.2/11 Max 15.4	3480
6	19 May ± 4.3/11	31 May ± 4.3/12	12.8 ± 1.8/11	12.8 ± 2.1/11	73.0 ± 6.8/11	5.7 ± 2.0/11 Max 8.4	2980
spring	28 Mar ± 5.5/14	31 May ± 4.3/12	43.0 ± 3.6/11	63.8 ± 7.0/12	270.8 ± 16.5/11	6.3 ± 2.9/11 Max 15.4	11 140

## 2. Methods

### (a) Study system and method details

Adult red-backed shrikes were caught and equipped with multisensor data loggers in their breeding territories in Gribskov, Denmark (55.98°N, 12.33°E), using mist nets or spring traps, during May–July, 2014–2018. The multisensor data loggers were attached to the birds as a backpack using leg-loop harness and the mass of the data logger plus attachment material was <1.2 g (3.6–4.7% of the body mass of the birds). In this study, data from 11 male and 4 female individuals are included. 12 of these 15 individuals were recaptured and the data logger retrieved 1 year after attachment, and three individuals were recaptured and the data logger retrieved 2 years after attachment (whereof two individuals had data for two full annual cycles and one individual had data for one full annual cycle and half of the second autumn). Because of the decrease in data quality during the second year, and to avoid pseudo-replication, only data from the first year are included in this study, but the actograms for these repeated journeys can be found in the electronic supplementary material, figure S1.

The multisensor data loggers used in this study included an accelerometer, a temperature-compensated barometric pressure sensor (recording both barometric pressure and temperature; not included in data loggers attached before 2016), a light-level sensor, a real-time clock and a memory [20,28]. In this study, we focused on the accelerometer data, allowing us to monitor in detail the activity of the birds throughout the full annual cycle. Depending on the programming of the data loggers, light was only recorded during five periods for 5 days throughout the year, and thus, light data were not sufficient to identify the stopover locations of the birds. However, when possible, we used the barometric data to determine the location of longer stopovers to identify the geographic area of each flight segment (barometric data were not available for all individuals, either depending on logger design or sensor failure; see §2c). The temperature data are not equal to ambient temperature [29] but are used to calibrate the barometric sensor and were not included in this study.

The accelerometer measured acceleration on a single axis (approximately vertical when the bird is flying). One sample was recorded every 5 min and the data were stored per hour (12 samples per hour). Each sample consisted of 5 subsamples (100 ms duration at 100 Hz) with 5 s in between. If all sub-samples detected activity (acceleration varied more than one-quarter g) then the sample scored ‘5’. If no subsamples detected activity, the sample scored ‘0’. Intermediate cases scored ‘1’ – ‘4’. Sample scores of 4 and 5 reflect birds in continuous flapping flight. For a detailed description of the function and measurement scheme of the multisensor data logger, see [20].

### (b) Identifying flights and flight segments

In the red-backed shrikes, continuous flight was identified based on the activity data when consecutive activity samples scored 4 or 5 [20]. Migratory flights were identified as periods longer than 30 min (minimum of 7 activity scores of ‘4’–‘5’ per hour) with continuous flight, and flight duration per each migratory flight was calculated from the sum of the samples during a night with activity score 4 or 5, as previously described in [20]. If a bird performed two flight periods during one night, this is regarded as two parts of one flight (or one night of flying).

Migratory flight segments were identified as periods when the shrikes performed clustered migratory flights. The migratory flight segments were separated by major stopovers (during autumn migration, these were classed as stops >9 days; during spring migration, this was taken as the longest stopover), except for the last two flight segments during spring migration. These two segments were distinguished based on a very distinct shift in course (Zugknick) that occurred in the Middle East after the birds had crossed the desert areas of the Arabian Peninsula by flying straight north and abruptly changing to W–NW courses when continuing from the Middle East towards Europe. We separated these periods into two segments as this distinct shift in course could be expected to be part of the birds' migratory programme, and in addition, because behavioural differences could be expected as the two segments are defined by large differences in habitat (the first includes crossing of the Arabian Desert and the second covers the temperate zone in Europe) and varying intensity in diurnal activity has previously been observed [22]. In most birds (7 out of 11 individuals), these two segments were not separated by a stopover, but the birds performed migratory flights on every consecutive night from the Horn of Africa to their breeding site in Denmark. Still, the abrupt change of course from N to NW was readily identifiable from the sudden delay in starting time for the nocturnal flights that appeared in the actogram when the birds started to fly on more westerly courses (experiencing later sunsets) into temperate Europe, with later and shorter nights. When nocturnal flights started more than 1.5 h later than the departure time for the first nocturnal flights from the Horn of Africa, the bird was considered to have entered Europe and initiated its sixth and last segment of its annual cycle. The abrupt course change signifying the transition between segments five and six was confirmed by the geographic analyses for the birds where it was possible to estimate location from barometric pressure even for short stops <24 h (see §2c; figure 2). The division between segments five and six was robust and remained the same independently of whether it was based on the departure time (activity data) or the geographic location of the abrupt course change (barometric pressure data). Thus, three main segments were identified during autumn migration (segments 1−3 in the annual cycle) and three further segments during spring migration (segments 4−6).

In addition to flights within the identified flight segments, most individuals performed isolated flights that were separated by at least 9 days from a migratory flight segment within longer stopovers or the wintering period. These flights are categorized as odd flights [20], which are not included in the flight segments, and hence they are also excluded from general analyses regarding the flight schedule, number of flights, flight durations and segment durations. However, figure 1 illustrates the entire annual activity pattern of the birds, including the odd flights.

### (c) Estimating geographical areas

To identify the geographical region of each flight segment, we used the R package GeoPressureR (v. 3.4.0; [27]), following the steps outlined in [30], by analysing the barometric pressure data when these were available for 9 individuals, of which barometric data only allowed for positioning during autumn migration for three individuals. Consequently, we first labelled the pressure and activity timeseries to define flight and stopover events. Second, we matched the measured barometric pressure variation with global reference weather data (ERA5 hourly at ground level; 0.5° × 0.5° grid size [31]) to create likelihood maps of positions for each stationary period for each individual [32]. Third, the likelihood maps were used to model the trajectory that maximizes the overall probability between the estimated stationary positions for each bird, taking flight duration (rounded to the nearest full hour; only flights ≥2 h were included) and a gamma-distributed flight speed distribution (minimum flight speed 20 km h^−1^; max flight speed 150 km h^−1^) into account. For each bird, we calculated the most likely path and the marginal probability for each stopover. From the most likely path, we calculated the migration distance between each stopover and the total distance covered for each segment. As we did not have continuous light data, the positioning was based solely on barometric pressure and activity data. For details on geolocation from pressure data, see [33].

For 6 out of 15 birds, the logger either did not include a pressure sensor, or there was a technical problem either with the pressure sensor or the clock of the logger. As previous studies have reported small variation between individuals both in temporal and spatial patterns [21], and the geographical stopover areas identified by analysing barometric pressure data did not deviate from this pattern, we are confident that each flight segment (see §2b) is assigned to the correct geographical area for all birds.

### (d) Statistical analysis

Numbers of flights and flight duration were calculated manually for each migratory segment. The duration of the segment was calculated from the day of the first flight within the segment until the day of the end of the last flight of the segment. All statistics were performed with R v.4.2.2 [34] and the descriptive statistics for all variables were calculated using the psych package [35]. Levene’s tests of heterogeneity were performed to analyse the differences in variation in flight numbers, flight duration and segment duration, both between migratory seasons and between the different flight segments within each migratory season, using the car package for R [36].

## 3. Results

### (a) The general structure of migration

All red-backed shrikes performed their migration with a clearly structured flight pattern, including periods of aggregated nocturnal flights alternating with stationary periods (figure 1; table 1). All birds used a nocturnal flight schedule, and flights were only occasionally prolonged by a few hours into daytime during the flight segments across the Mediterranean Sea and the Sahara Desert during autumn migration, and during the flight segment across the Arabian Peninsula during spring migration (figure 1). The individual migratory flights varied in duration and lasted on average 6.2 ± 3.2 h (mean ± s.d.), and the longest recorded flight lasted 17.2 h (figure 1; table 1).

The red-backed shrikes departed from their breeding ground in Denmark at the end of July and, during their first flight segment, flew for approximately 33 flight hours, divided into approximately 8 flights, to reach their first major stopover site in southeastern Europe. They remained in this area for 18.6 ± 8.6 days (approximate distance 1420 km; table 1; figures 1 and 2). At the end of August, the birds departed from southeastern Europe and crossed the Mediterranean Sea and the Sahara Desert in *ca* 67 flight hours divided into *ca* 9 flights (approximate distance 3560 km; table 1; figures 1 and 2), before staying in Sahel for 49.2 ± 6.8 days. The birds departed on the third flight segment at the beginning of November, when they left Sahel to fly *ca* 90 h in *ca* 14 flights (table 1; figures 1 and 2) to their main winter area in southwestern Africa in the longest flight segment during autumn migration (approximate distance 4140 km). During autumn migration, the variance in segment duration, flight duration and number of flights between the three different flight segments did not differ (Levene’s test of heterogeneity within autumn migration; flight duration: *F*_2,41_ = 1.25, *p* = 0.30, segment duration *F*_2,41_ = 2.88, *p* = 0.068, flight numbers: *F*_2,41_ = 0.69, *p* = 0.51; figure 3). The birds arrived at their wintering grounds at the end of November and stayed for 120.6 ± 10.5 days before departing on spring migration at the end of March (table 1; figures 1 and 2).

During the first spring segment (segment 4; approximate distance 4680 km) the birds crossed Africa in *ca* 122 flight hours divided into *ca* 21 flights to the Horn of Africa in the longest flight segment of their migration (table 1; figures 1 and 2). The birds stayed 5.8 ± 3.5 days at the Horn of Africa before completing the last two flight segments back to the breeding area. After leaving the Horn of Africa, the birds flew north and crossed the Arabian Peninsula in *ca* 73 flight hours, divided into *ca* 9 flights (approximate distance 3480 km). Seven out of eleven birds did not stop in between segment 5 and segment 6, and of the remaining four individuals, three individuals stayed for only one day and the last individual for three days (average stopover 0.6 ± 0.9 days). In the last segment, the birds shifted to a north-westerly course after passing the Arabian Peninsula and travelled across eastern Europe back to their breeding grounds in Denmark in *ca* 73 flight hours divided into *ca* 13 flights (approximate distance 2980 km; table 1; figures 1 and 2). The last two segments (segments 5 and 6) were characterized by very few stops and very little variation between individuals (figures 1 and 3). All birds performed the last flight segment back to their breeding grounds with consecutive flights every night, and the nocturnal flight duration gradually decreased as the birds flew north and encountered shorter nights with increasing latitudes and an advancing spring season. The birds returned to their breeding grounds by the end of May (table 1). During spring, the last two segments (segments 5 and 6) varied less between individuals compared with the first spring segment (segment 4) regarding their flight durations, numbers of flights and segment durations (Levene’s test of heterogeneity within spring; flight duration: *F*_2,31_ = 9.09, *p* = 0.00078, flight numbers: *F*_2,31_ = 12.01, *p* = 0.00014, segment duration: *F*_2,31_ = 10.36, *p* = 0.00036; figure 3).

Total duration of migratory flight was, on average, 189 h for autumn migration (with coefficient of variation between individuals CV = 10 %) and 271 h (CV = 6%) for spring migration. These flight hours were completed in, on average, 31 flights (CV = 16%) during autumn and 43 flights (CV = 16%) in spring (figure 3). The variation in flight duration, number of flights and the duration of the seasonal migration did not differ between individuals during autumn and spring migrations (Levene’s test of heterogeneity between autumn and spring migration; flight duration: *F*_1,23_ = 0.044, *p* = 0.95, flight numbers: *F*_1,23_ = 0.31, *p* = 0.59, segment duration: *F*_1,24_ = 3.46, *p* = 0.075; figure 3).

### (b) Odd flights

All birds except one individual (for which data are only included for autumn migration) performed nocturnal flights outside the main migratory periods at some point throughout the annual cycle. Such odd flights were recorded during four phases of the annual cycle: before the migratory period in autumn, within the autumn stopover period in eastern Europe, within the autumn stopover period in the Sahel region and during the winter period in southern Africa (electronic supplementary material, Table S1). On average, all birds had a total flight duration of 459.3 h divided into 73.5 migratory flights for the full annual cycle, whereof odd flights made up, on average, 13.5 ± 10.5 flight hours divided into 3.3 ± 2.4 flights. Hence, the odd flights comprised only 2.9% of total flight duration and 4.3% of the total number of flights. On average, these flights were relatively short and lasted for 4.4 ± 2.1 h (for basic statistics per stationary phase, see electronic supplementary material, Table S1).

## 4. Discussion

### (a) Characteristics and variations of migratory flights

To our knowledge, this is the first study of annual flight activity in a migratory songbird that provides a more extensive sample size and thus makes it possible to, at least to some extent, analyse the variation in the organization of the migratory flight activity between individuals. All red-backed shrikes performed a migration with an annual flight pattern that was highly structured, with characteristic aggregations of flights during successive segments that could be readily identified for all individuals. There was generally a high degree of consistency between individuals in this annual flight pattern, as indicated by the low variability in flight characteristics, both within the different segments and for total autumn and spring migration (figures 1–3; table 1). The relative variation (CV; figure 3) between individuals was in several cases remarkably small, falling below 20%. These results are in agreement with previous studies of this population that have observed low inter-individual variability in routes and timing of migration [21–23]. Yet, the phenology of the different flight segments during autumn migration, and especially the initiation of autumn migration, varied considerably between individuals (figure 2), possibly as an effect of differences in breeding success between the different individuals.

Throughout their migration, the red-backed shrikes used a nocturnal flight schedule with only extremely few exceptions when flights were prolonged into daytime, possibly in connection with water crossings (as indicated by the estimated positioning and the timing of the flights). Such exceptional prolonged flights were recorded about once per individual. However, these flights were prolonged by only a few hours into daytime and never continued throughout a full day (figure 1). This indicates a clearly lower propensity to fly into daytime compared with what has been reported from studies of barrier crossing strategies in several other songbird species (e.g. [37–40]). That the propensity to perform prolonged flights into daytime differs noticeably between species indicates that this is most probably based on the birds’ internal migratory programme and potentially related to the biology and/or migratory route of the species and/or population.

Besides the regular migratory flights, almost all birds performed odd flights that were excluded from the six identified flight segments (see §2). The total flight duration for odd flights was, on average, 13.5 h per bird, which is equivalent to 2.9% of the total annual flight time. These odd flights predominantly took place in the middle of long stationary periods and were commonly initiated in the middle of the night, rather than at dusk (figure 1). Although the exact nature of these types of flights is unknown, it is possible to speculate that they are a response to adjustments in the stopover location owing to decreased food availability and/or competition for territories outside the breeding areas [41]. However, since the odd flights accounted for only a very minor part of the total annual flight activity, they have a negligible influence on the overall structure of the annual flight activity.

### (b) Actograms for migrants in cages and in the wild

When obtaining the first annual actograms for the red-backed shrikes, we were very surprised to see how the pattern of nocturnal flight activity was radically different from our expectations based on the recordings of migratory restlessness among caged migrants [9,10], including the red-backed shrike [27]. Actograms of caged migrants showed that migratory restlessness occurred on most nights, indicating a preparedness for migratory flights on the majority of nights during the autumn and spring migration seasons. Thus, we expected that migratory flights from free-flying birds would be rather uniformly spread throughout the autumn and spring migratory periods, with some short-term variation depending on variable local and regional conditions for flight and fuel deposition. Hence, the detailed flight patterns during a migratory season were expected to differ significantly between individuals and seasons, depending on the shifting short-term conditions for flight and fuelling.

In glaring contrast to these expectations, the actograms of free-flying red-backed shrikes showed that the patterns of migratory flight were highly structured, with flights occurring in aggregations or clusters that made it possible to subdivide the total migratory journey into a series of segments. Six distinctive flight segments were recognized for the annual migration cycle of the red-backed shrike, and these segments were identified for all individuals (*n* = 15) and years (*n* = 4) in this study. Most of these segments were separated by long stopover periods, but in one case, different segments were separated based on an abrupt and distinct change in flight course (segments 5 and 6; see §2b). Each segment has its characteristic actogram ‘barcode’ pattern, albeit with some variation in timing and flights as presented in table 1 and figures 1–3. It seems clear that the migratory travelling plan for the red-backed shrikes is much more carefully organized than suggested by the more schematic patterns of restlessness as revealed by the experiments with caged migrants.

The difference between migratory flight activity from the first published actogram of a free-flying red-backed shrike [20] and the migratory restlessness of the caged conspecific [27] has been highlighted previously [13–15]. A closer comparison between the patterns of migratory restlessness (birds in cages) and of migratory flights in the wild of red-backed shrikes reported in this study reveals some discrepancies that are interesting but difficult to explain. Both birds in cages and in the wild had their main autumn migratory period in August–November (four months), but while the total duration of restlessness was on average 310−320 h for caged shrikes from French and Finnish populations [27], the total migratory flight time during the autumn period for the free-flying Danish shrikes was only on average 190 h (max 220 h; table 1, figure 3). It seems that the total restlessness time for a migration period may represent a possible maximum potential limit of flight activity rather than directly determining the distance/flight duration of migration.

It should be noted that we have recorded actograms of migratory flight patterns for adult birds (retrieval of loggers not possible for first-year shrikes because of their low degree of natal philopatry) that belonged to a different population from the birds that were studied in cages. We think that it is still valid to consider the actograms of adults in relation to the internal programme of migratory restlessness, because it seems very likely that the adults' journeys reflect their preceding journeys as yearlings to a high degree. The changes and improvements after the first trip must, after all, build on the main characteristics of the first trip, and succeeding trips cannot therefore deviate too much in their main migratory flight structure from the first journey. Similarly, as red-backed shrikes from several European populations have been observed to show similar patterns in their migratory routes and timing [42,43], we do not believe that the Finnish bird would differ significantly from the Scandinavian population.

Among caged shrikes, the intensity of migratory restlessness reached its highest levels at the beginning of the autumn migration period. About 60% of the total interval of restlessness during the autumn period occurred during August and September, before the birds steadily decreased their activity throughout the rest of the migratory period [27]. This does not fit with the distribution of flight time among the free-flying shrikes. They slowly increased their cumulative flight hours during the first two flight segments in August and September (figure 2) and showed a clear peak in activity during the third segment in November (table 1, figures 1–3; see also Fig. 1 in [22]). Between those peaks in activity, the free-living shrikes had a long stationary period of about two months during their autumn migration season from the beginning of September to the beginning of November, before starting their third, long segment across the African continent towards southern Africa (table 1, figure 3). It is surprising that there was no indication whatsoever of a dip towards zero when considering the migratory restlessness of caged shrikes during this long stationary period [27].

There are as yet only very few studies showing the distribution of migratory flights during the annual migration cycle. Accelerometer studies of few individuals of lesser grey shrike *Lanius minor* [44] and barred warbler *Sylvia nisoria* [45]—both species with a geographic loop migration pattern similar (although less extensive) to that of the red-backed shrike—indicate a well-defined structure with nocturnal migratory flights organized into segments, as for the red-backed shrike. Such a structured distribution of annual migratory flight activity also seems to apply to the Chilean elaenia *Elaenia chilensis*, migrating in the South American continent between Patagonian breeding grounds and non-breeding areas in Brazil [46]. For the barred warbler, there are also actograms of the migratory restlessness of caged individuals [47] for comparison, which reveals similar discrepancies to those found for the red-backed shrike. The total time of autumn migratory restlessness was about 250 h for the caged birds, while the total flight time for autumn migration in two free-flying individuals was clearly lower than this, at 140 and 168 h, respectively [45,47]. Restlessness intensity was highest in the first 1−2 months of the autumn migration period, which coincides with frequent migratory flights across Europe, the Middle East and Sahara, while the late within-Africa migration was less extensive. The long stationary period during more than two months in Sudan, before the barred warblers continued to their final non-breeding sites, did not appear at all in the actograms of the caged warblers.

More variable is the flight actogram of the tawny pipit *Anthus campestris*, which migrates a shorter distance than the abovementioned species and furthermore carries out its migratory flights not only during the nights but also in the early morning hours [48]. This indicates that the detailed control of the migratory travel plan may differ between long- and short-distance migrants, nocturnal and diurnal migrants, and between species and populations with migratory journeys of different complexity, etc.

### (c) How could the realized structure of migration be regulated?

Given the observed annual flight patterns of the red-backed shrikes, as manifested in the actograms in figure 1, how is this complex behaviour with its consistent aggregations of migratory flights and its segmental structure controlled? Weather and fuelling conditions are expected to be quite variable for different individuals at different sites, years and times (the 15 individuals included in this study performed their migration during four different years and with different timings; figure 2d). Hence, we consider it unlikely that the consistent structure of migration that we observed in these free-flying birds, with clusters of migratory flights instead of the expected rather uniformly spread flights throughout migratory periods, is a response only to variation in weather, winds and local fuel deposition conditions en route. Thus, there is probably an internal/genetic flight plan that controls these key features of the flight patterns for the annual migratory journey. One may speculate that the control of the migratory programme depends on an interaction between an internal/genetic flight plan and external cues that are necessary for the proper successive stepwise advancement of the migratory journey.

When a free-flying bird records critical specific cues (geomagnetic, celestial, olfactory, etc.), its internal flight plan will make it react in a specific way at a specific latitude/position. This type of migratory signpost or marker has been discussed previously for migratory songbirds (e.g. [49–52]) and is further described and explored in sea turtles [53], Pacific salmon [54] and European eels [55]. In migratory birds, a magnetic map has been suggested to guide navigation in experienced individuals (e.g. [56–58]), but caged juvenile songbirds have also been shown to react in a biologically relevant way to an artificial displacement in geomagnetic space, indicating that magnetic signposts might play a role in affecting the first migration as well. Juvenile thrush nightingales *Luscinia luscinia* have been shown to increase their fuel load when magnetically displaced to the expected Mediterranean stopover before the flight segment across the Sahara Desert compared with control birds experiencing the natural magnetic field in central Sweden [49]. However, late in the season, when the control birds were still experiencing the magnetic conditions of central Sweden and were expected to be stressed owing to their delayed timing, the difference between the groups disappeared, indicating a magnetic effect on both timing and fuelling [51]. Similarly, late in the season, juvenile European robins (*Erithacus rubecula)* increased their fuel load when exposed to the local magnetic field of central Sweden in comparison with birds magnetically displaced to non-breeding areas in Spain [50]. Migratory activity has also been shown to decrease in juvenile Northern wheatears *Oenanthe oenanthe* that were magnetically displaced to their non-breeding sites in Western Africa compared with birds exposed to the natural magnetic field in Germany during autumn [59]. Altogether, these studies indicate that geomagnetic cues could play a significant role not only for orientation and navigation in birds but also as signposts guiding the migratory behaviour of birds along their migratory journey and potentially forming part of the migratory flight plan together with the bird’s internal programme.

## 5. Conclusions

This study demonstrates that the migratory flights of long-distance migratory red-backed shrikes are not scattered along the flyway but aggregated into flight segments that can be recognized for all individuals. The structure is more apparent during autumn compared with spring, as the shrikes perform a very condensed spring migration with relatively few stops overall (on average 270 flight hours divided on 43 flights over a two-month period; table 1; figures 1 and 2). There is a pronounced difference between this clearly segmented migration showed by free-flying birds and the expectations from earlier studies on caged migrants that flights would occur regularly throughout the migratory season reflecting near-constant migratory restlessness during migratory periods. In contrast, the free-flying birds showed highly consistent and predictable clusters of migratory flights and longer stopovers between flight segments. It is clear that the structure of migration that we now, with the access to new technology, can observe in free-flying migratory birds demands a more intricate regulation than has previously been imagined. This regulation is likely to involve more than the total amount of migratory restlessness rather evenly distributed during the migratory season, but also the amount of flight hours in clusters of migratory flights during specific segments of the migratory journey. Potentially, this regulation is based on an internal migratory programme that acts in combination with a complex feedback mechanism system involving external signals (magnetic, celestial, photoperiod cues, etc.).

## Data Availability

All raw data (including data for the analyses for geolocation by pressure and figure 2c) and the data and code for all analyses and figures 1, 2a,b and d and 3 are available at Dryad [60]. All code and data for the analyses for geolocation by pressure and figure 2c are available at Zenodo: [61] Supplementary material is available online [62].
